# Impact of Coronary Microvascular Dysfunction in Takotsubo Syndrome: Cause, Consequence or Both?

**DOI:** 10.31083/j.rcm2505163

**Published:** 2024-05-11

**Authors:** Serena Caglioni, Daniela Mele, Andrea Milzi, Luca Bergamaschi, Anna Giulia Pavon, Antonio Landi

**Affiliations:** ^1^Cardiocentro Ticino Institute, Ente Ospedaliero Cantonale (EOC), CH-6900 Lugano, Switzerland; ^2^Cardiology Unit, Azienda Ospedaliera Universitaria di Ferrara, 44124 Cona, Italy; ^3^Cardiology Unit, IRCCS Galeazzi, Sant’Ambrogio Hospital, 20157 Milan, Italy; ^4^Cardiology Unit, IRCCS Sant’Orsola-Malpighi Hospital, 40138 Bologna, Italy; ^5^Department of Medical and Surgical Sciences -DIMEC, University of Bologna, 40138 Bologna, Italy; ^6^Department of Biomedical Sciences, University of Italian Switzerland, 6900 Lugano, Switzerland

**Keywords:** Takotsubo syndrome, pathophysiology, microcirculation, coronary microvascular dysfunction

## Abstract

Takotsubo syndrome (TTS) is an acute cause of heart failure characterized by a reversible left ventricular (LV) impairment usually induced by a physical or emotional trigger. TTS is not always a benign disease since it is associated with a relatively higher risk of life-threatening complications, such as cardiogenic shock, ventricular arrhythmias, respiratory failure, cardiopulmonary resuscitation and death. Despite notable advancements in the management of patients with TTS, physiopathological mechanisms underlying transient LV dysfunction remain largely unknown. Since TTS carries similar prognostic implications than acute myocardial infarction, the identification of mechanisms and predictors of worse prognosis remain key to establish appropriate treatments. The greater prevalence of TTS among post-menopausal women and the activation of the neuro-cardiac axis triggered by physical or emotional stressors paved the way forward to several studies focused on coronary microcirculation and impaired blood flow as the main physiopathological mechanisms of TTS. However, whether microvascular dysfunction is the cause or a consequence of transient LV impairment remains still unsettled. This review provides an up-to-date summary of available evidence supporting the role of microvascular dysfunction in TTS pathogenesis, summarizing contemporary invasive and non-invasive diagnostic techniques for its assessment. We will also discuss novel techniques focused on microvascular dysfunction in TTS which may support clinicians for the implementation of tailored treatments.

## 1. Introduction

Takotsubo syndrome (TTS) is an acute cause of heart failure characterized by transient left ventricular (LV) dysfunction, usually triggered by emotional or physical stressors, that account for approximately 1–3% of patients with suspected acute myocardial infarction (AMI) [1]. When female patients with suspected AMI are separately appraised, its frequency rises up to 5–6% [1]. Post-menopausal women account for up to the 90% of TTS subjects [2]. TTS is not always a benign disease since several studies have shown similar prognostic implications than AMI [2, 3, 4]. Up to 10% of patients with TTS have an annualized higher risk of major adverse cardiac and cerebrovascular events [2].

Several mechanisms have been proposed in the TTS pathophysiology, but the exact pathway connecting myocardium, nervous system, systemic vasculature and circulating amines is still lacking. Coronary microvascular dysfunction (CMD) is an increasing recognized entity which has been advocated in the pathophysiology of TTS [5]. However, whether CMD represents an epiphenomenon or the precipitating cause of TTS is still matter of debate. The scope of the present review is to provide an update on TTS pathophysiology with a special focus on the emerging role of CMD. We will also provide a summary of novel invasive and non-invasive techniques to identify CMD in TTS patients.

## 2. Physiopatological Mechanisms of TTS

**Fig. 1.**
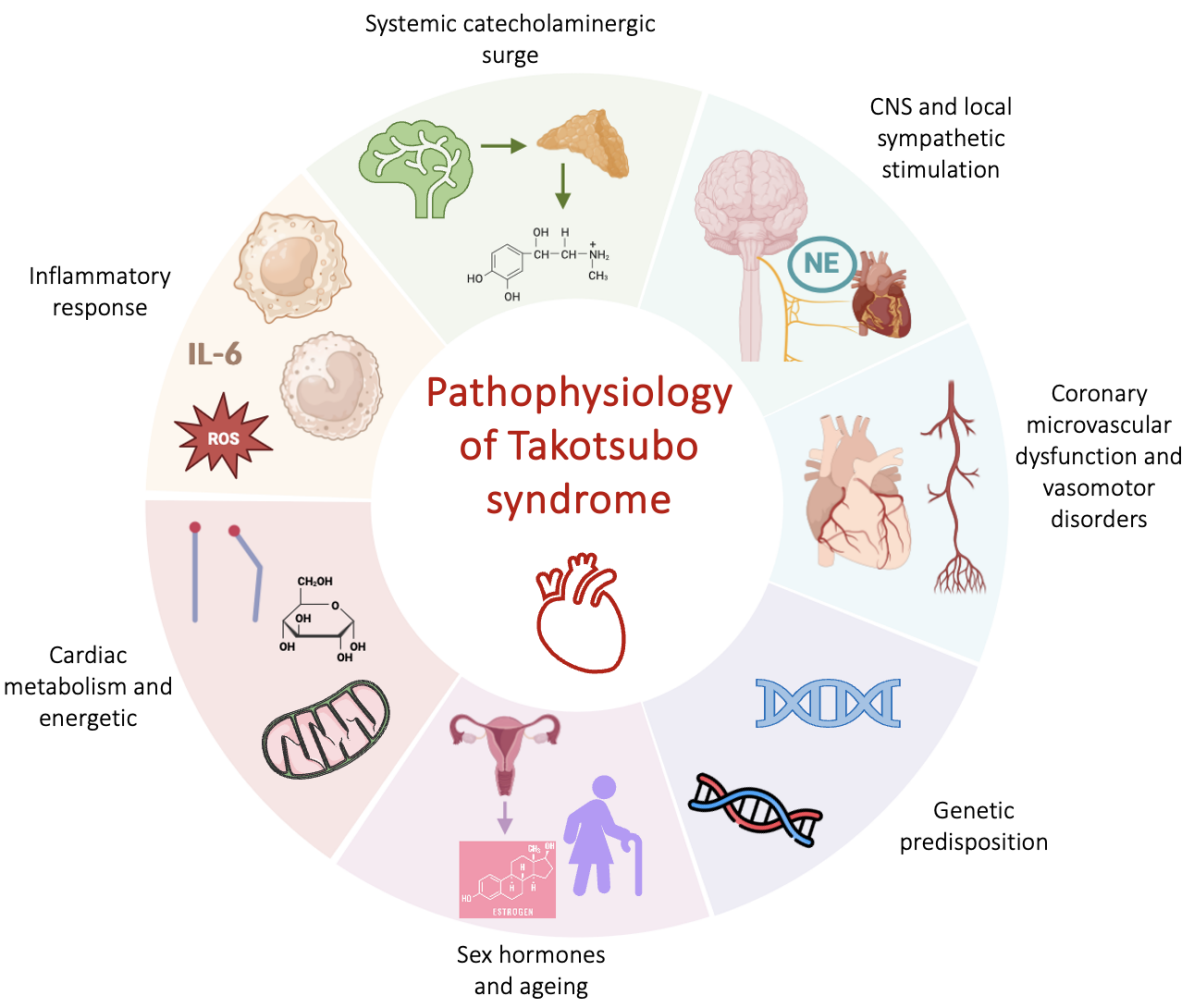
**Physiopathological mechanisms of TTS**. Abbreviations: CNS, central nervous system; NE, norepinephrine; ROS, reactive oxygen species; TTS, takotsubo syndrome; IL-6, interleukin 6.

The exact pathophysiological mechanism behind transient LV dysfunction is still unsettled. Despite TTS resembles for some aspects an AMI, other mechanisms rather than cardiomyocyte necrosis are involved, as documented by the limited troponin elevation and lack of late gadolinium enhancement (LGE) at cardiac magnetic resonance (CMR) [1]. Several hypotheses have emerged to explain the unique features of this disease (Fig. 1). Among them, the catecholaminergic theory, based on an increase in systemic or local catecholamines is the most accredited one. There is consolidated evidence demonstrating the detrimental effects of catecholamines excess in both human and pre-clinical models. High levels of serum catecholamines in patients with pheochromocytoma can induce LV regional wall-motion abnormalities similarly as in TTS [6, 7] and also exogenous administration of adrenaline or dobutamine in humans is associated with the development of the syndrome [8]. High systemic and local levels of catecholamines have been found in the acute phase of TTS, with plasma values that resulted greater compared with subjects with heart failure due to AMI [9, 10]. Histopathological observations of contraction band necrosis in biopsies from patients with TTS further support the sympathetic theory [9]. This is a peculiar form of myocyte injury consisting in contracted sarcomeres, eosinophilic bands and mononuclear inflammatory infiltrates, that are normally observed in presence of catecholamine excess, such as pheochromocytoma or acute neurological illness [11, 12]. A transient form of LV dysfunction, that sometimes spares the apex region, has been described in several acute cerebrovascular diseases such as subarachnoid haemorrhage (SAH), highlighting the link between neurovascular events and the genesis of TTS [13]. Additionally, in preclinical models, intravenous administration of epinephrine, norepinephrine, dobutamine or isoprenaline has proven to induce a reversible Takotsubo-like cardiac dysfunction [14, 15, 16, 17]. The description of TTS in transplanted hearts or in those with chronic spinal cord transection above the level at which the heart sympathetic fibers leave the spinal cord, do not support the hypothesis of catecholamine local release [18, 19].

The clinical presentation of TTS seems not consistent with a catecholamine surge, since hypertensive crisis or sinus tachycardia are relatively uncommon. Furthermore, there are conflicting data regarding the increase in systemic catecholamines, with a recent study showing normal levels [20]. Since different patterns of regional wall motion abnormalities have been described, local distribution of the adrenergic receptors within the myocardium could explain the different patterns of TTS. In mammalian heart models β1 and β2 adrenoreceptor density showed a gradient from the apex (more represented) to the base, suggesting a potential higher susceptibility of the apical and mid segments to the circulating catecholamines [21]. The sympathetic innervation of the left ventricle, in contrast, is higher at the base compared to the apex and this could hypothetically explain a neuro-mediated form of reverse TTS, in which the basal regions are involved [22, 23].

The mechanisms whereby catecholamine excess acts at myocardium level causing LV stunning is another controversial aspect. Epinephrine and norepinephrine normally improve cardiomyocytes contractility binding β1 and β2 adrenoreceptors (AR), activating the G stimulatory (Gs) protein family and consequently increasing the intracellular calcium. β2AR are less represented in the myocardium compared to β1 and, at variance with the latter, are linked to both Gs and G inhibitory (Gi) proteins [16]. Supra-physiological concentrations of epinephrine, after binding β2AR and coupling to the Gi proteins, have shown inhibitory activities leading to a negative inotropic effect, which may be prevented via Gi inactivation by pertussis toxin pre-treatment [16]. Additionally, the β2AR-Gi activation determines an antiapoptotic cardioprotective effect [16, 17, 24]. The predominant density of β2AR at the apical level supports the physiopathological basis of apical ballooning in TTS but not the other atypical TTS phenotypes. A transient myocardial dysfunction, known as neurocardiogenic stunning (NS), is a well-recognized condition following acute central nervous system injury (e.g., SAH or stroke), affecting predominantly the basal and mid-ventricular segments [25]. These observations allow us to speculate that NS and TTS are two sides of the same coin of the catecholamine-mediated myocardial effect through two different ways. While apical ballooning could be explained by a systemic increase in catecholamine levels, basal and mid ventricular patterns of NS may be justified by neuro-mediated local release of norepinephrine. These findings are in line with the higher prevalence of neurologic or psychiatric disorders among TTS than AMI patients, observed in the InterTAK registry [2]. The same group demonstrated a hypoconnectivity of central brain regions related to autonomic functions and regulation of the limbic system in acute TTS phase compared to controls, further supporting the role of the brain-heart interaction in TTS pathogenesis [26].

Several reports described the occurrence of TTS among family members [27, 28, 29, 30]. Genetic polymorphisms of β1and β2 adrenoreceptor have been inconsistently associated with myocardial stunning after a SAH [31, 32, 33]. Additionally, the rs17098707 polymorphism in the G protein-coupled receptor kinase 5 gene, implicated in the intracellular pathway of β adrenoreceptors signalling, has shown to carry a higher risk of TTS [34]. A larger study characterizing the genotype of TTS subjects is currently ongoing to definitely ascertain a potential role of genetic predisposition in TTS (GENETIC [Is There a Genetic Predisposition for Acute Stress-induced {Takotsubo}Cardiomyopathy], NCT04513054).

There is increasing evidence supporting the role of local and systemic inflammation in the acute and chronic phase of TTS. A recent multicentre study demonstrated an intramyocardial macrophage infiltrate during the acute phase using ultrasmall superparamagnetic particle of iron oxide enhanced CMR, in both affected and not affected LV, which was no longer detectable at follow-up [35]. Additionally, some studies demonstrated a sustained inflammatory response in TTS patients as documented by the increase in serum interleukine-6, chemokine (C-X-C motif) ligand one and classic cluster of differentiation (CD) 14^++^CD16^––^ monocytes [35]. From a clinical standpoint, the occurrence of heart failure might be explained by this low-grade, chronic inflammatory substrate [36]. Differently from other cardiac conditions, such as myocarditis, macrophages are the main component of the inflammatory infiltrate of TTS, with a preponderance of proinflammatory M1 than M2 type [37, 38].

The impaired cardiac metabolism and energetics found in preclinical models of TTS can also have a role in the pathogenesis of the disease [39].

TTS is one of the cardiovascular disorders with the most pronounced gender difference, since up to 90% of the affected subjects are women [2]. There is increasing evidence suggesting that supplementation of oestrogens is able to mitigate the stress-induced LV dysfunction in a rat model and oestradiol seemed to have a protective effect against the excess of catecholamines on cardiomyocytes [40, 41]. Despite this preclinical evidence, no difference in oestrogens plasma levels has been documented between patients with TTS and AMI. In addition, the presence of hormone replacement therapy in postmenopausal women doesn’t seem to have a protective role against the occurrence of TTS [42, 43]. On the basis of the peculiar epidemiology of TTS, its relationship with gender and sex hormones deserves further investigations.

The vascular system has been also advocated as one of the main players in the pathogenesis of TTS. At the very beginning, a spontaneous multivessel epicardial spasm was described during invasive coronary angiography and consequently advocated as the mechanism responsible of the observed LV-dysfunction [44]. This hypothesis is little supported by evidences, due to the lack of reproducibility of this pioneering finding in subsequent reports and, additionally, epicardial coronary spasm hardly would justified the non-coronary distribution of the akinetic regions. CMD is an increasingly recognize entity that has been reported in several cardiovascular diseases, especially in myocardial infarction without obstructive coronary artery disease (MINOCA) [45]. Reversible myocardial perfusion defects and CMD, were extensively demonstrated in the acute phase of TTS using both invasive and non-invasive techniques [45, 46, 47, 48, 49, 50, 51, 52, 53, 54, 55]. Whether CMD has a causative role or represent a secondary phenomenon, triggered by myocardial inflammation and oedema, remains to be entirely established. The apparently increased vascular reactivity and decreased endothelial function in patients with a previous TTS episode might suggest a vasomotor dysfunction as a potential precipitating cause of TTS [56]. Preclinical evidence, in which the normalization of myocardial perfusion restores its function, seems to support this hypothesis [55, 57]. An attempt to address this question was done in a continuously monitored rat preclinical model, in which no detectable perfusion defects preceded the isoproterenol induced apical ballooning [58], making CMD most likely a consequence rather than the cause of TTS. Several mechanisms could potentially explain the microcirculatory impairment as a secondary phenomenon: (i) the inflammatory infiltrate and oedema described in the myocardial akinetic segments; (ii) the decreased relaxation of involved regions, being the myocardial perfusion mainly a diastolic process, and (iii) the connection between cardiac metabolic demand, that is expected to be reduced in the affected myocardium, and perfusion provided by autoregulatory mechanisms [23, 59].

A comprehensive appraisal of the mechanisms underlying TTS would help to address an appropriate treatment, that represents the major unmet need in this scenario.

## 3. Coronary Microvascular Dysfunction

CMD encompass a large spectrum of structural and/or functional microcirculatory conditions that determines an impairment in coronary blood flow resulting in a myocardial demand-supply mismatch. Architectural changes within microcirculation such as vascular smooth muscle hypertrophy, capillary rarefaction, perivascular fibrosis, together with endothelium-dependent or independent vasomotor dysfunction contribute to the development of CMD [60]. Given the established role of microcirculation in different cardiovascular diseases, several invasive and non-invasive techniques have been developed for coronary microvascular function assessment as summarized in Table 1.

**Table 1.** **Invasive and non-invasive diagnostic techniques for CMD**.

	Measure	Technique	Formula	Specific for micro-circulation
Invasive Techniques	Coronary flow reserve (CFR)	Bolus/continuous thermodilution or intracoronary Doppler	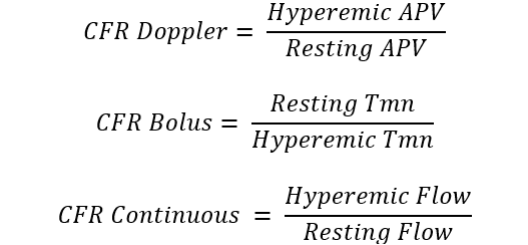	No
	Index of microcirculatory resistance (IMR)	Bolus thermodilution	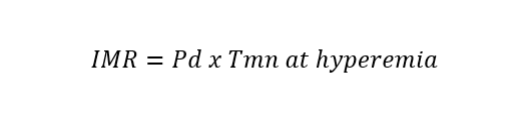	Yes
	Hyperaemic microvascular resistance index (HMR)	Intracoronary Doppler	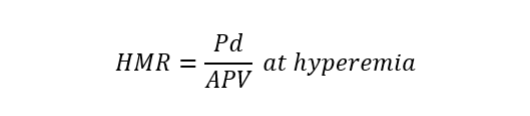	Yes
	Microvascular resistance (Rµ)	Continuous thermodilution	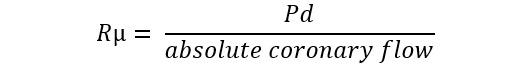	Yes
	Microvascular resistance reserve (MRR)	Continuous thermodilution, potentially also with other techniques	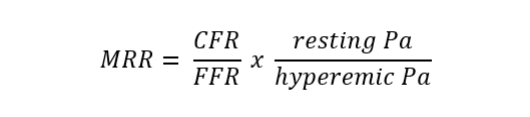	Yes
	Angio-derived IMR	Computation of coronary flow velocity from angiography	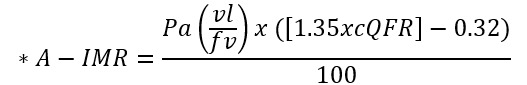	Yes
Non-Invasive Techniques	Coronary flow velocity ratio (CFVR)	Transthoracic Doppler echocardiography	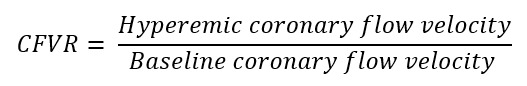	No
	Myocardial perfusion reserve (MPR)	PET, CMR, contrast echocardiography	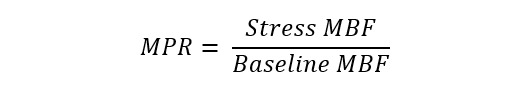	No
	Myocardial perfusion reserve index (MPRI)	CMR, CT	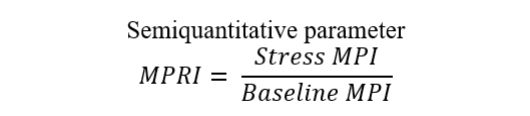	No

*Different formulae are provided for the calculation of angio-derived IMR. Abbreviations: APV, average peak velocity; Tmn, mean transit time; Pa, aortic pressure; vl, vessel length; fv, flow velocity; cQFR, contrast quantitative flow ratio; PET, positron emission tomography; CMD, coronary microvascular dysfunction; CMR, cardiac magnetic resonance; MBF, myocardial blood flow; CT, computed tomography; MPI, myocardial perfusion index; Pd, distal coronary pressure; FFR, fractional flow reserve.

### 3.1 CMD by Non-Invasive Techniques

Several non-invasive imaging modalities are utilized in the assessment of CMD and could be useful in the work-up of patients with TTS [61, 62]. Non-invasive techniques are able to evaluate, through different methods, the vasodilatory response of the coronary microcirculation but, differently from invasive methods, they do not allow to test the tendency of coronary arteries to spasm [63].

Transthoracic echocardiography (TTE) is usually the first-line imaging technique applied in TTS patients, due to its large availability and the possibility to be performed bed-side [64]. TTE enables the detection of CMD through either Doppler technique or contrast echocardiography [61, 65]. A study conducted by Galiuto *et al*. [46] utilized contrast echocardiography to show that patients with TTS exhibited reversible apical perfusion defects following adenosine infusion. This study demonstrated an acute and reversible coronary microvascular impairment in subjects with apical TTS, by showing that segments with dysfunctional wall motion had lower myocardial blood flow (MBF) velocity and MBF [46]. The existence of CMD can be also assessed by Doppler TTE evaluating on left descending coronary artery the coronary flow reserve (CFR) dividing stress peak coronary flow velocity by the resting one [61]. In thirty TTS patients the evaluation of CFR through Doppler TTE was feasible and showed an impaired value upon admission (1.8 ± 0.2) with a progressive recovery in the sub-acute phase at discharge [66]. However, it must be noticed that CFR measured by TTE can be challenging and strongly depends on patient’s acoustic window. Therefore, CFR by Doppler TTE is not routinely evaluated in clinical practice [67].

Nuclear medicine techniques represent the non-invasive gold-standard for evaluation of non-endothelial dependent microvascular function in absence of obstructive coronary artery disease (CAD) by measuring absolute MBF and MBF reserve [68]. Small studies have demonstrated minor perfusion abnormalities in patients with TTS by 18-fluorodeoxyglucose (FDG) positron emission tomography (PET) or single-photon emission computed tomography (SPECT) imaging [69]. Nuclear medicine imaging has proven its worth also in giving insight about the physiopathology of TTS, showing an “inverse metabolic perfusion mismatch” characterized by an impaired metabolism in the involved LV regions with normal MBF at rest [70, 71, 72].

CMR and cardiac computed tomography angiography (CCTA) can also be used in the TTS diagnostic work-up [61, 62, 64]. CMR is able to overcome some limitations of poor TTE acoustic window and can be very useful in the subacute phase [73]. At CMR, the microcirculation can be assessed by employing the myocardial perfusion reserve index (MPRI) as a semiquantitative parameter that reflects the vasodilatory capacity of small blood vessels [61, 62]. The MPRI is defined as the ratio of stress to rest upslope normalized to the upslope of the LV blood pool [74]. However, to date, the evaluation of CMD by CMR remains underutilized in clinical practice, especially in TTS patients. There is increasing evidence suggesting that CMD may affect myocardial perfusion during hyperemia [75]. Thus far, only high-resolution CMR has been associated with good accuracy in quantitatively detecting CMD [76].

Recent advances in CMR and CCTA technology now also afford to serially imaging the transit of the contrast (gadolinium or nonionic iodine) in the arterial circulation and in the myocardium and quantification of MBF in milliliters per minute can also apply per gram as described for PET imaging.

Semi-quantitative evaluation of resting and hyperemic myocardial perfusion is feasible by static computed tomography (CT) perfusion (CTP) and recently, the presence of impaired myocardial perfusion in women with angina and no obstructive CAD was demonstrated by CT-CPT [77, 78].

In order to assess properly the results of non-invasive imaging modalities, the presence of obstructive CAD should be excluded through invasive coronary angiography or CCTA. From this perspective, CCTA could become a useful tool in the assessment of TTS patients, giving its well-established role in rule out significant CAD and the potential information provided about myocardial perfusion.

### 3.2 CMD by Invasive Techniques

The main parameter used to detect CMD by invasive techniques is the ratio between hyperaemic and resting coronary flow, named CFR [79]. This proportion represents the capacity of coronary flow to increase following a hyperaemic stimulus, mainly consisting of adenosine administration, that simulate the physiologic response to efforts [79]. Typically coronary blood flow is able to increase at least 2-times and consequently the normal CFR value is above 2 or 2.5, depending on the implemented methodology [79]. Two surrogates of flow can be used in clinical practice to calculate CFR: coronary flow velocity and mean transit time of a room-temperature saline bolus. The former is measured by a dedicated wire with a pressure-Doppler sensor while the latter technique evaluates the saline bolus mean transit time through a pressure-temperature wire by thermodilution principles. The Doppler method is technically more challenging, due to the difficulty in obtaining good velocity Doppler signals [80]. On the other hand, bolus thermodilution is highly operator-dependent, given the manual rapid injections required, characterized by large intraobserver variability [81]. Using both techniques, hyperaemic values are divided by baseline values to obtain CFR and CMD can be defined based on CFR (<2 with Doppler or <2.5 with bolus thermodilution) only in the absence of coronary epicardial disease, being this index potentially influenced by both micro and macro-circulation. The index of microcirculatory resistance (IMR) was proposed to overcome this limitation as a metric specific for the microcirculation, defined as the product between distal coronary pressure and mean transit time of a 3-cc saline bolus during steady-state hyperaemia. An IMR value equal or greater than 25 is suggestive of CMD [82].

Recently, a method measuring absolute coronary blood flow based on continuous thermodilution principle has emerged [83, 84]. This quantitative approach is completely operator-independent and allow to directly assess the resting and hyperaemic flow (mL/min) and microvascular resistance (WU) by a continuous coronary infusion of saline through a dedicated monorail microcatheter. The ratio between true baseline and hyperaemic microvascular resistance defined the microvascular resistance reserve (MRR) which is a new attractive microvasculature specific metric to quantify CMD [85]. CFR and MRR derived from continuous thermodilution resulted significantly lower and showed higher repeatability compared to CFR and MRR obtained with bolus thermodilution [86].

All the techniques described above required dedicated and expensive tools (i.e., guidewires with specific sensors, microcatheters) and the administration of vasodilator agents, resulting in a longer procedural time. A novel metric specific for the microcirculation directly derived from angiography, named angio-derived IMR has been also developed [87]. Several formulae with a superimposed diagnostic performance have been proposed to calculate angio-derived IMR [47], characterized by an overall high diagnostic accuracy (AUC 0.86) in assessing CMD when compared to wire-based IMR [88, 89].

A comprehensive full physiology approach for CMD includes also the evaluation of coronary vasomotor function through specific provocative tests [90]. The agents commonly used in clinical practice to test the coronary endothelium-dependent vasomotion function are acetylcholine (ACh) and ergonovine. While in the healthy endothelium ACh mediates the production of nitric oxide (NO), a potent vasodilator, in the presence of endothelial dysfunction (ED) it is able to trigger a paradoxical epicardial or microvascular vasoconstriction [90]. While the epicardial spasm is easily recognized in the angiographic images following increasing doses of ACh, given the inability to visualize directly the microvascular bed, its vasoconstriction is suggested by the concomitant occurrence of chest pain and ischemic electrocardiographic changes in the absence of epicardial spasm. The presence of abnormal endothelium-dependent vasoreactivity, consisting of coronary vasospasm induced by ACh, was reported in up to 85% of patients with TTS during the acute phase [91].

**Fig. 2.**
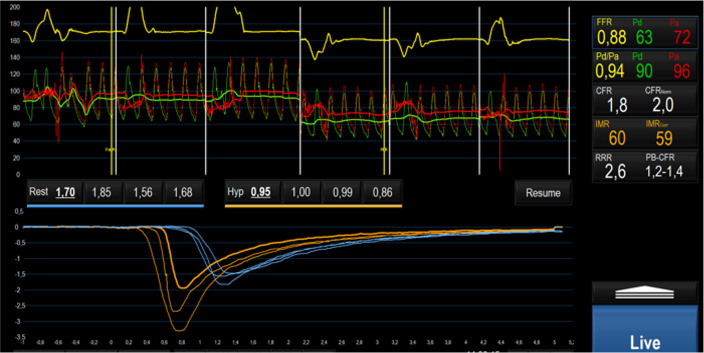
**Invasive assessment of CMD on left anterior descending artery through bolus thermodilution in a patient during the acute phase of TTS, characterized by high IMR and low CFR values**. Abbreviations: CMD, coronary microvascular dysfunction; FFR, fractional flow reserve; Pd, distal pressure; Pa, aortic pressure; CFR, coronary flow reserve; IMR, index of microcirculatory resistance; RRR, resistive reserve ratio; TTS, takotsubo syndrome; Hyp, Hyperemia; PB-CFR, pressure bounded coronary flow reserve.

In TTS patients undergoing coronary angiography, retrospective evaluation of angio-derived IMR confirmed the presence of microvascular dysfunction in at least one coronary vessel [45, 47, 92]. Angio-IMR values were inversely correlated with LV function and associated with higher N-terminal pro B-type natruretic peptide (NT-pro-BNP) levels, implying a connection between the degree of microvascular and myocardial dysfunction [92]. In TTS angio-IMR was not significantly higher, compared to the other forms of MINOCA, in which a microvascular impairment has been also documented [45]. Small prospective studies and several case reports further acknowledge the microvascular dysfunction, defined in terms of IMR and CFR derived from bolus thermodilution, as a key feature of TTS; an example is depicted in Fig. 2 [48, 50, 51, 52, 93]. In 20 female patients with TTS, concomitant measure of IMR and inflammatory mediators from aorta and coronary sinus samples confirmed the presence of high levels of inflammatory biomarkers without showing any correlation with IMR values [52]. Recently, a comprehensive invasive assessment with both bolus and continuous thermodilution in the acute TTS phase, reported the presence of CMD, characterized by high microvascular resistance and low coronary flow during the steady-state hyperaemia. CMD as well as LV function showed a recovery at the 3 months follow-up [94]. The demonstration of the transient nature of CMD is more challenging, due to the risk at which the patient would be exposed in case of a systematic reassessment of microcirculation. However, the normalization of microvascular function at one or three months follow-up has been reported in small patient cohorts [93, 94].

## 4. Evidence Supporting the Role of CMD in TTS

The potential impact of CMD in the complex pathogenesis of TTS is presented in the following paragraphs.

### 4.1 Catecholamine-Induced Transient LV Dysfunction and CMD

An overactivation of sympathetic drive remains one of the most accredited physiopathological hypothesis of transient CMD as a result of catecholamines effects on vascular α adrenoreceptors or of direct toxic myocyte injury. Recently, in a murine model, Dong *et al*. [55] demonstrated an altered flow regulation in the apex before development of TTS-like phenotype. In addition, the restoration of perfusion, through coronary vasodilator or via genetic re-expression of a $K^{+}$ channel involved in coronary flow regulation, determined a normalization in the LV function. These findings support the pivotal role of CMD in the pathophysiology of TTS and a strategy aimed at restoring the MBF, such as the use of coronary vasodilator, might represent a potential therapeutic target. On the other hand, Redfors *et al*. [58] failed to demonstrate the presence of myocardial perfusion defects preceding a isoproterenol induced apical ballooning in a rat model, without evidence at the biopsies of microvascular structural damage.

In humans, an acute myocardial perfusion defect has been documented through contrast echocardiography in the stunned myocardial regions and this alteration, differently from AMI, slightly improved as LV function recovers after adenosine infusion [46]. Thus, a transient coronary microvascular constriction, completely recovered at 1 month follow-up, which could be induced after a stressful event by catecholamines, could represent another potential pathogenetic pathway [46].

The precise role played by coronary microcirculation in the pathogenesis of TTS and its relationship with catecholamines, remains matter of investigation, albeit its involvement as a key feature of the syndrome is unquestionable.

### 4.2 Crosstalk between Hormonal Variations and Endothelial Function

Post-menopausal older women are typically affected by TTS and the risk of developing the disease increases about five times in females >55 years old, compared to younger ones [95]. An enhanced activity of the sympathetic nervous system is associated with progressive ageing, regardless of gender, potentially contributing to the increased incidence of TTS with older age [96]. Additionally, in post-menopausal women, vagal cardiac tone seems to decrease [97]. The switch between the two components of the autonomous nervous system, with a prevalence of the sympathetic over the vagal tone in postmenopausal women, could predispose to TTS. Oestrogens have a protective role against the development of cardiovascular diseases in women and probably, also against the occurrence of TTS. The way through sex hormones explicate this effect has been extensively investigated and one possible explanation might reside in their effect on stress response. Oestradiol supplementation in perimenopausal women attenuates the response to mental stress in terms of blood pressure and release of cortisol, adrenocorticotropic hormone (ACTH), plasma epinephrine and norepinephrine [98]. Another significant aspect in the pathogenesis of TTS is the link between sex hormones, ED and CMD [99]. There is increasing evidence that ED is a key aspect of TTS both during the acute and long-term phase [56, 57, 100]. The endothelium is the main determinant of vascular tone, through the production of vasodilatory and vasoconstrictive substances and its function can be influenced by sex hormones with either receptor-dependent or independent mechanisms, thanks to the direct expression of oestrogens receptors on human vascular endothelium and smooth muscle cells [101, 102]. Oestrogens are vasoactive hormones, able to upregulate the synthesis of NO, one of the most potent vasodilators in a receptor-mediated manner and physiologic oestrogen levels in postmenopausal women can potentiate the endothelium-dependent coronary and systemic vasodilatation [103, 104, 105]. The endothelium shows an age-related dysfunction, as documented by the progressive loss of systemic flow mediated dilatator capacity, which differs across sexes [106]. In addition, the documented relationship between endothelial-dependent vasomotion in systemic (i.e., brachial) and coronary arteries, supports an interplay between ED and CMD, that are probably two faces of the same coin [107].

## 5. Knowledge Gaps

Despite the increasing awareness of TTS as a transient heart failure syndrome and the advancements in its diagnostic processes, the precise pathophysiological mechanisms remain matter of further investigation. Different hypotheses have emerged to explain the unique course of the disease and recently, the involvement of coronary microcirculation, has gained popularity.

The uncertainties regarding the exact pathophysiological process at the basis of the disease, is probably the main reason behind the lack of validated therapeutic options. Currently no evidence-based therapy exists for TTS either in the acute phase of the disease or at long-term, characterized by significant morbidity and mortality. Randomized controlled clinical trials are still ongoing to investigate different therapeutic options in TTS, including the use of apixaban for the prevention of thromboembolic complications [108, 109]. Future large-scale studies are warranted to better understand this unique disease and to identify novel therapeutic targets.

## 6. Conclusions

TTS represents a peculiar cardiovascular syndrome characterized by a transient myocardial dysfunction, usually precipitates by emotional or physical triggers. Despite its apparent benign nature, TTS is associated with a significant morbidity and mortality, comparable to acute coronary syndromes. Sex hormonal variations and their effect on endothelial function can predispose to the development of TTS. Enhanced activity of sympathetic nervous system and CMD play a crucial role in the pathophysiology of the disease, although the exact pathway involved remains matter of further investigations. Whether CMD could represent a potential therapeutic target in the acute phase of TTS is worthy of future research.
